# ENDO-ERN expert opinion on the differential diagnosis of pubertal delay

**DOI:** 10.1007/s12020-021-02626-z

**Published:** 2021-01-29

**Authors:** Luca Persani, Marco Bonomi, Martine Cools, Mehul Dattani, Leo Dunkel, Claus H. Gravholt, Anders Juul

**Affiliations:** 1 Department of Medical Biotechnology and Translational Medicine, University of Milan, Milan, Italy; 2 Department of Endocrine and Metabolic Diseases, IRCCS Istituto Auxologico Italiano, Milan, Italy; 3 Department of Internal Medicine and Pediatrics, Ghent University and Pediatric Endocrinology Service, Ghent University Hospital, Ghent, Belgium; 4 Genetics and Genomic Medicine Research and Teaching Programme, UCL GOS Institute of Child Health, London, UK; 5 Department of Endocrinology, Great Ormond Street Hospital for Children, London, UK; 6 Centre for Endocrinology, William Harvey Research Institute, Barts and the London School of Medicine and Dentistry, Queen Mary University of London, EC1M 6BQ London, UK; 7 Department of Endocrinology, Aarhus University Hospital, Aarhus, Denmark; 8 Department of Molecular Medicine, Aarhus University Hospital, Aarhus, Denmark; 9 Department of Growth and Reproduction, University of Copenhagen, Rigshospitalet, Denmark

**Keywords:** Hypogonadism, Pubertal delay, Constitutional delay of growth and puberty, Gonadal dysgenesis, Primary ovarian insufficiency, Sex steroid priming

## Abstract

The differential diagnoses of pubertal delay include hypergonadotropic hypogonadism and congenital hypogonadotropic hypogonadism (CHH), as well as constitutional delay of growth and puberty (CDGP). Distinguishing between CDGP and CHH may be challenging, and the scientific community has been struggling to develop diagnostic tests that allow an accurate differential diagnosis. Indeed, an adequate and timely management is critical in order to enable optimal clinical and psychosocial outcomes of the different forms of pubertal delays. In this review, we provide an updated insight on the differential diagnoses of pubertal delay, including the available tests, their meanings and accuracy, as well as some clues to effectively orientate towards either constitutional pubertal delay or pathologic CHH and hypergonadotropic hypogonadism.

## Delay of puberty

### The process of puberty and its delay

Physiological pubertal development encompasses a sequential series of pubertal stages, usually occurring between the age of 8 and 13 years in females, and 9 and 14 years in males. The first signs of pubertal development are represented by testicular enlargement (testicular volume >3 mL) in males and breast budding (thelarche) in females. The appearance of pubic and axillary hair is mainly dependent upon adrenal androgens and, if isolated, should not be interpreted as the beginning of gonadotrophin-dependent puberty [1, 2]. However, in some ethnic populations pubarche may be the first sign of pubertal onset in a large proportion of children.

The mechanisms involved in the timing of puberty are complex and currently not fully understood, but several intrinsic (e.g. genetic) and environmental cues are involved in this process [3, 4].

Pubertal delay refers to the absence of the first signs of pubertal development beyond the normal age distribution (>2–2.5 standard deviation above the mean of the reference population) or, when pubertal progress arrests after an initial physiological start [5]. A considerable delay in pubertal onset is associated with significant somatic and psychological impact on the adolescent [6].

### Causes of delayed puberty

Delay of puberty is very common. Constitutional delay of growth and puberty (CDGP) is the most frequent form and is a paraphysiological self-limited condition. Highly variable conditions include the various causes of primary and central hypogonadism (Table 1).

**Table 1:** List of conditions associated with pubertal delay

	Para-physiological condition	Disease conditions		
Frequency	CDGP (self-limited) 1:50	Hypogonadotropic hypogonadism		Hypergonadotropic hypogonadism 1:600–2000
		Genetic or Organic 1:5000–20,000	Functional (reversible) variable	
	Common familial component: − Frequent autosomal dominant inheritance (associated genes: *IGSF10*, *HS6ST1, EAP1, LGR4, FTO*)	− CHH (Kallmann or nCHH) (many genes with variable inheritance and expressivity) − CHARGE and other genetic syndromes (eg, Prader Willi, UMS) − MPHD (several genes with variable inheritance) − Acquired lesions (e.g. tumors, infiltrative lesions) − Thalassemia − Iatrogenic causes (e.g. surgery, radiotherapy)	− Chronic diseases − Malabsorption − Malnutrition − Anorexia nervosa − Excessive exercise − Stress − Drugs − Other endocrine diseases (e.g. hyper-PRL)	− Klinefelter or Turner syndromes − Gonadal dysgenesis − Enzymatic defects − LH/FSH resistance − Acquired forms (e.g. chemo- or radio-therapy; autoimmune diseases; traumas)

## Hypergonadotropic hypogonadism

In hypergonadotropic hypogonadism the central compartment of the HPG axis activates normally with the onset of puberty, but the gonads do not respond appropriately due to an intrinsic gonadal defect. Hypergonadotropic hypogonadism can be differentiated from other forms of pubertal delay through medical history, physical examination, and particularly unstimulated reproductive hormone levels, characterized by low sex steroids and high gonadotropins (Table 2). Medical and family history should focus on co-morbidities, e.g. auto-immunity, or previous oncological treatment. In girls, a prepubertal uterus can be very difficult to visualize by ultrasound, and caution should be made not to interpret this as congenital absence of a uterus (e.g. due to Mayer–Rokitansi syndrome). For this reason, we recommend that these ultrasounds should be conducted and interpreted by an experienced specialist. Karyotyping may reveal an atypical sex chromosomal constitution, such as 45,X in a girl or 47,XXY in a boy. More detailed genetic investigations, mostly by whole exome or genome sequencing, complement the diagnostic work-up in hypergonadotropic hypogonadism, and may reveal conditions like differences/disorders of sex development (DSD) or primary ovarian insufficiency (POI). Genetics underlying DSD has been reviewed recently [7].

**Table 2:** Clinical differences between various forms of pubertal delay

	Constitutional delay of growth and puberty (CDGP)	Congenital hypogonadotropic hypogonadism (CHH)	Primary gonadal defects (e.g., Turner/Klinefelter syndromes, others)
Sex prevalence	Males	Males	Males
Growth velocity	Prepubertal, concordant with bone age	Prepubertal	Prepubertal, severely impaired in Turner syndrome (TS)
Pubertal status	Prepubertal at presentation	Prepubertal in complete form; partial or arrested development possible	Prepubertal if complete form; partial or arrested development possible
Bone Age in adolescents	Delayed by 1–3 years	Frequently delayed by 1–3 years	Variable delay; uncommon delay in Klinefelter syndrome (KS)
Predicted adult height (PAH)	Lower limit of target height (TH)	>TH (if untreated)	Often >TH (if untreated) in KS; <TH in TS
Past history illness	Generally silent; may be associated with current or previous chronic disease or atopy	“Red flags”: cryptorchidism (typically bilateral), micropenis, renal anomalies, midline defects, hypo/anosmia, hearing loss, …	“Red flags”: short stature and other syndromic stigmata in TS; mental retardation/learning difficulties in KS; atypical genitalia in gonadal defects; specific syndromic features in ovarian dysgenesis
Family history (FH)	70–80% positive for CDGP; possible history of syndromic conditions associated with self-limited pubertal delay (eg. ulnar mammary syndrome…)	Frequent family history of self-limited delayed puberty or CHH. or both. FH of anosmia/bimanual synkinesia/midline or hearing defects and others	Frequently negative (mostly associated with infertility), but asymptomatic carrier status and phenotypic heterogeneity are common
Gonadotropins	Inappropriately low	Inappropriately low	High

## Central hypogonadism

Central or hypogonadotropic hypogonadism includes hormonal disturbances that may be due to either organic (acquired or congenital) or functional (such as chronic illnesses, obsessive focus on food, lifestyle and exercise/orthorexia, or frank anorexia requiring nutritional rehabilitation) conditions that affect either the pituitary and/or the hypothalamus. Due to the common isolated presentation, the most frequent differential diagnoses are CDGP and congenital hypogonadotropic hypogonadism (CHH), which can be either normosmic (nCHH) or associated with an olfactory defect, i.e. Kallmann syndrome (KS) [8]. The pathogenesis of both forms of CHH is still frequently undefined, although a genetic background can be determined in ~50% of cases, and the diagnostic yield is likely to increase in the near future [9, 10].

CDGP refers to a paraphysiological, self-limited form of delayed puberty that usually represents an extreme of the normal spectrum of pubertal timing. It is the most common cause of pubertal delay and in clinical settings much more common in boys than girls [5]. The significant familial recurrence of this defect is indicative of a strong genetic predisposition. Although the inheritance of CDGP is variable, an autosomal dominant pattern, in association with environmental modifiers, has been frequently observed, with a complete or incomplete penetrance. So far, no specific causal genes have been identified to account for the majority of cases of CDGP [11]. Patients with CDGP may experience slower growth rate, which results in a lower final adult height [1], delayed sexual maturation and delayed skeletal age compared to peers. Usually, the condition has a good prognosis with full completion of pubertal maturity.

CDGP can be diagnosed only after exclusion of pathologically absent puberty such as hypergonadotropic hypogonadism or CHH. However, differentiating between CDGP and CHH can be extremely challenging in adolescence. Leaving the diagnosis for later in life, though easier, may have negative consequences on adult height, and is frequently the cause of low self-esteem and behavioral problems and should therefore be avoided [12].

Once systemic pathologies determining functional HH are ruled out on clinical examination, the differential diagnosis between these two conditions is not straightforward (Table 2). Adolescents with CHH and CDGP both have similar clinical features and hormone profiles, i.e., low gonadotropins (FSH, LH) and sex steroids (testosterone in males and estradiol in females). Studies on the discriminatory value of Sertoli cell markers (e.g., inhibin B and AMH) or GnRH and hCG stimulation tests have not proven to be truly reliable [13, 14].

### Clinical features

Pubertal delay in males is diagnosed when the testicular volume, using Prader’s orchidometer, has not reached 4 mL by the age of 14 years [8, 15]. Some characteristic features may serve as “red flags”, as their presence may point towards a specific diagnosis or a diagnostic subgroup (Table 2) [5]. Features of CHH include olfactory defects, clefting of the palate and/or lips, hearing loss, alteration of digital bones, colorblindness, nystagmus and bi-manual synkinesia [16–18]. Renal malformations are often present in CHH, so a renal ultrasound should be included to rule out a morphogenetic defect.

Family history can be positive for delayed puberty in both CDGP and CHH, even with overlap between the two conditions within the same family, making the differential diagnosis even harder [19, 20]. Nevertheless, it is important to investigate, given the well-documented familial recurrence of self-limited pubertal delay or CHH, as well as of its characteristic-related features (i.e., anosmia, renal malformations, hearing loss, color blindness, etc.).

In boys, the presence of neonatal cryptorchidism (particularly the bilateral forms) and micropenis are important findings, pointing to a prenatal and postnatal gonadotropin deficiency. All boys with an underdeveloped (at least 2.5 SD below the mean) but otherwise normal penis, without hypospadias, should be further investigated to exclude CHH, based on baseline assessment of the HPG axis during minipuberty (~1–4 months after birth), or an HCG stimulation test thereafter, due to the physiological reduction of gonadotropins until puberty [2, 21]. More complex forms of genital under-virilization are more likely due to hypergonadotropic hypogonadism rather than CHH [6, 22]. Unfortunately, no specific signs may point to CHH in girls during the neonatal period and throughout childhood.

Boys and girls with CHH experience normal growth during childhood, and the absence of long-bone epiphyseal closure explains their frequent eunuchoid aspect and relative tallness when diagnosed and thus treatment is delayed. Retarded bone maturation, osteopenia, and osteoporosis are often found when the diagnosis of CHH is made later in life [23, 24].

Short stature with slow growth velocity and low weight can all point towards functional forms of HH [15] (Table 1). When these features are mild and syndromic manifestations are absent, and particularly if there is a positive family history, the most likely diagnosis is CDGP.

Although in most CHH patients, puberty never occurs, adult-onset forms of heritable HH exist [25]. These men typically complain of low libido and erectile dysfunction. In patients with isolated GnRH deficiency the finding of a partial progression through puberty followed by a permanent arrest of sexual maturation is not uncommon, which makes the differentiation between the two conditions even more difficult [26].

Olfactory defects should be evaluated with a quantitative olfactory test and/or MRI of the olfactory structures; however, it should be noted that defects of smell affect <50% of subjects with CHH. Still, whenever present, olfactory defects should be considered a reliable clue towards this diagnosis [27].

### Hormones and stimulation tests

An important feature of CHH is the low gonadotropin concentrations in the pre-pubertal range. However, this feature alone cannot differentiate between CDGP and CHH in early adolescence, as gonadotropin concentrations are frequently bordeline even in healthy subjects (Table 3).

**Table 3:** Elements for the differential diagnosis between CDGP and CHH in males

	CDGP	CHH
Pubertal delay	Yes, but transient	Yes, but permanent
Syndromic features	Uncommon	Common (e.g. anosmia, midline defects)
Bone age	Retarded	Retarded
Testo/E2	Low	Low
LH/FSH	Inappropriately low	Inappropriately low
GnRH test	Blunted/appropriate response	Absent/blunted response
AMH, Inhibin B, INSL3	Low-normal	Low
HCG stimulation	Normal Testosterone rise	Often subnormal testosterone rise
Genetic studies	*IGSF10* and HS6ST1 monoallelic variants + wild-type classic CHH genes (ie. GnRHR, TAC3, TACR3, HS6ST1)	Pathogenic variants in candidate genes, oligogenic involvement
Sex hormone priming for 3–6 months: − Testo enanthate: 50–100 mg/month − Testo gel: 10 mg every 2 days → 1 day	Primed pubertal start: ↑ bi-testicular volume >8 mL	Absent pubertal start: HH persistence and bi-testicular volume <8 mL

To differentiate between CHH and CDGP, the study of LH nocturnal pulses has been proposed: in fact, the lack of nocturnal LH pulses in adolescence was described to be specific for HH, but a similar pulsatile fluctuation was found in prepubertal children and patients with CDGP and CHH by using ultrasensitive assays [28]. The only difference between the two conditions seems to be the absence of entrainment to nocturnal sleep and the low incidence of synchronization between LH and FSH pulses [28, 29]. However, the analysis of the nocturnal patterns of gonadotropins cannot be used as a diagnostic test in routine clinical practice.

In order to avoid nocturnal sampling, the diagnostic utility of a single basal gonadotropin concentration has been proposed, and a threshold of LH concentrations above 0.2 U/L would indicate onset of puberty [5, 30], but the accuracy is very low. A similar diagnostic power can be assigned to the dynamic gonadotropin testing with GnRH or GnRH analogs. The use of GnRH agonists (GnRHa) seemed to have a higher discriminatory potential compared to GnRH for dynamic testing [31]. However, the absence of a gonadotropin response to GnRH stimulation can confirm the absence of puberty onset, rather than a true differential diagnosis between CDGP and CHH (Table 3). The determination of urinary gonadotropins can be a valuable perspective to be validated in this context [32].

Other endocrine markers have been proposed to guide the differential diagnosis. These include the functional measurement of Sertoli cells (inhibin B), immature Sertoli cells (AMH), and Leydig cells (INSL3 and testosterone after hCG stimulation) [33]. Though promising in certain cases [31, 34, 35] the available data are insufficient to clearly identify the potential clinical utility of these markers mainly due to the overlap of values between CDGP and CHH. The availability of new methods with higher sensitivity/specificity for their determination may open novel perspectives (e.g. LC/MS approach).

The measurement of serum AMH concentrations combined with inhibin B has proven to give reassuring results in cases with a clinical suspicion of pubertal disorders, since their concentrations reflect the function and number of Sertoli cells. While these tests cannot discriminate with guaranteed accuracy between CHH and CDGP they can, however, be helpful in the early detection of testicular tubular damage [36].

The testosterone response to a longer-term (19 days) HCG stimulation test was found to give significantly lower testosterone levels in CHH, as were the peak serum FSH responses to GnRH stimulation [37] (Table 3).

However, the combination of several tests is too cumbersome and expensive to be used as a first line investigational approach.

Among the new frontiers of hormonal evaluation, the responsiveness to the neuropeptide kisspeptin has been investigated in boys with delayed puberty: Chan Y.M. et al. hypothesized in 2018 that kisspeptin would stimulate LH secretion in healthy subjects but not in CHH. At this point, further studies are needed to see if the kisspeptin stimulation test in prepubertal children can accurately predict outcomes for younger boys presenting with delayed puberty, since the published study included 17 years old patients, and our main priority is to correctly diagnose correctly the condition in boys and girls in their young teenage years, around the onset of physiological puberty [38].

### Genetics

To date, 60 genes are known to be associated with the multiple forms of CHH [9], all of them being genes that regulate development, migration and secretory function of GnRH neurons, but there is less knowledge regarding the genetic background of CDGP (Table 3). However, CDGP clusters in families [39] and a further study revealed a degree of overlap between the genetics of CHH and CDGP [20]. These findings were confirmed in a recent study describing a somewhat shared genetic predisposition, even if a greater prevalence of CHH variants and oligogenic involvement were more typical of CHH patients [40]. Nevertheless, the main utility of contemporary genetic studies remains its use in family counseling once the diagnosis is established, rather than as a routine first-line test.

### Priming

Another means to investigate pubertal delay, is the so-called low dose sex steroid (LDSS) “priming” test (Table 3). Once partial idiopathic GH deficiency has been excluded as a possible cause of slow growth rate (normal IGF1 and GH response to primed stimulation), this option can be proposed with the intrinsic advantage of also being the treatment of choice in CDGP. In fact, recent studies have investigated the diagnostic utility of testosterone priming not only to treat CDGP, but also to differentiate this condition from CHH. This is based on the idea that short-term testosterone treatment (injections, oral, or transdermal) in boys with CDGP would trigger HPG activation, with enlargement of the testis and increase in endogenous production of testosterone as a result [41]. One of the advantages of this interventional approach is a shorter delay in the diagnosis and consequent treatment, since the “diagnostic test” is also a treatment, with both psychological and physical benefits on growth rate and virilization. Very recently, a retrospective study performed by three ENDO-ERN centers reported that LDSS priming was associated with a significant improvement in final height of CDGP patients [42].

As a result, more expensive tests could be reserved for those who do not start puberty even after testosterone priming (i.e. CHH patients) [41]. On the other hand, it has to be considered that it is currently not known if testosterone pretreatment prior to gonadotropin therapy could affect the future fertility potential of male CHH patients [43–45]. Side effects of testosterone priming (including acne and in a minority of cases also aggressive behavior and painful erections) have been shown to be dose-dependent [46].

In any case, although testosterone priming cannot be considered an “unsafe” treatment, patients and their family need to be informed about the connected risks, and most importantly, in case of priapism a urologist has to be consulted in a timely manner [47].

The same approach has been attempted in females using low doses of estradiol as a priming therapy. The current evidence in females, however, is based mostly on girls who have β-Thalassemia, of whom 60–80% manifest hypogonadotropic hypogonadism, suggesting that the estradiol priming could be as useful as testosterone priming in the awakening of the hypothalamo-pituitary axis in CDGP female patients [48].

The data also suggest that sex steroid priming could increase the discriminatory power of dynamic tests (such as gonadotropin or GH stimulation) if given before such tests, indicating an interesting application of the technique in terms of the differential diagnosis of CDGP [42].

## Conclusions: How can one approach the differential diagnosis?

**Fig. 1:**
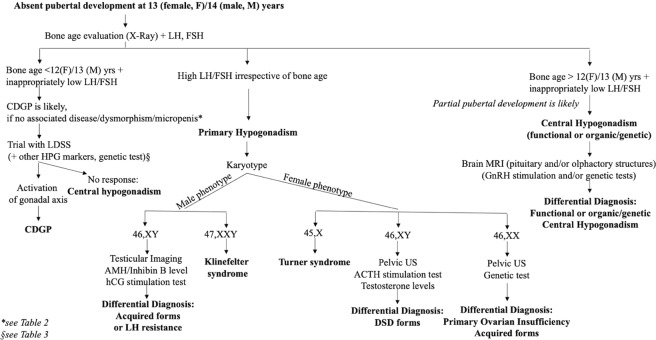
Diagnostic flow chart for delayed puberty. CDGP constitutional delay of growth and puberty, LDSS low dose sex steroid, HPG axis hypothalamus–pituitary–gonadal axis, US ultrasound

From the above it is clear that there is no strong and unequivocal evidence to guide us in the differential diagnosis and management of CDGP and CHH. However, according to the pathophysiology of these two conditions, we propose a tentative flow-chart that could lead through this tangle of tests and approaches (Fig. 1).

First of all, girls and boys with pubertal delay who do not have any of the typically associated features can be reassured before 13 or 14 years, respectively [8, 48, 49], as these ages are considered the upper limit of physiological puberty. If no sign of puberty has emerged at these ages, or if puberty does not progress appropriately according to puberty nomograms for girls and boys, respectively [50, 51], the determination of gonadotropins and bone age can be useful as a first diagnostic approach. High gonadotropins point to a primary gonadal defect and karyotype is a key step for the definition of these cases. In the case of gonadotropins that are inappropriately low for the hypogonadal state a significant bone age delay is indicative of a complete pubertal immaturity and in the absence of particular phenotypes a CDGP is the most likely condition. Based on the above reported data and the potential benefits for psychological wellbeing and final height [42], LDSS priming should be proposed to promote a jump start of spontaneous puberty in CDGP after a thorough discussion with the patient and their parents. Certainly, if differential diagnoses of CHH or hypergonadotropic hypogonadism are reached, appropriate steroid or gonadotropin treatment should be given in a timely manner at ages corresponding to physiological puberty in order to minimize psychological and somatic consequences.
